# Physicochemical Characterization and Larvicidal Activity of a Rutin–Zn Material against *Aedes aegypti*

**DOI:** 10.1021/acsomega.6c05197

**Published:** 2026-08-29

**Authors:** Daniel S. Santos, Robert S. Matos, Henrique D. da Fonseca Filho, Mário R. P. da Silva, Ricardo M. dos A. Ferreira, Raimundo N. P. Souto, José C. T. Carvalho, Erveton P. Pinto, Tiago M. de Souza, Irlon M. Ferreira, Caio P. Fernandes

**Affiliations:** † Departamento de Educação do Campo: Agronomia e Biologia, Universidade Federal do Amapá, Campus Mazagão, Mazagão, Amapá 68940-000, Brasil; ‡ Amazonian Materials Group, Department of Physics, 74364 Federal University of Amapá, Macapá, Amapá 68903-419, Brazil; § Laboratory for Development and Applications of Amazon Nanomaterials (LADENA) − Department of Materials Physics, 67892 Federal University of Amazonas, Manaus, Amazonas 69067-005, Brazil; ∥ Postgraduate Program in Materials Science and Engineering, Federal University of Sergipe-UFS, São Cristovão, Sergipe 49100-000, Brazil; ⊥ Departamento de Ciências Biológicas e da Saúde, 549483 Universidade Federal do Amapá, Campus Marco Zero, Macapá, Amapá 68903-419, Brasil; # Departamento de Ciências Exatas e Tecnológicas, Universidade Federal do Amapá, Campus Marco Zero, Macapá, Amapá 68903-419, Brasil; ∇ Departamento de Engenharia Química, Universidade Estadual do Amapá, Macapá, Amapá 68900-070, Brasil

## Abstract

Rutin is a natural flavonoid whose larvicidal activity may be limited by its physicochemical properties. Here, a rutin–Zn material was prepared using a nominal 1:1 precursor molar ratio and evaluated against fourth-instar *Aedes aegypti* larvae. UV–Vis spectroscopy showed shifts of Band II from 258 to 270 nm and Band I from 361 to 439 nm. FTIR bands assigned to O–H and aromatic/conjugated vibrations shifted from 3391 to 3163 cm^–1^ and from 1601 to 1560 cm^–1^, respectively, while a new band at 694 cm^–1^ was tentatively associated with a Zn–O vibration. NMR revealed perturbations in several ligand environments, including an apparent C-5 shift from 157.5 to 161.6 ppm, supporting interaction between Zn^2+^ and oxygenated regions of rutin without defining a unique coordination site or stoichiometry. X-ray diffraction indicated limited structural reorganization and residual precursor-related crystalline features. The intensity-weighted hydrodynamic diameter increased from 226 ± 3 nm for free rutin to 1501 ± 19 nm for the rutin–Zn material, indicating the formation of larger solvated aggregates under the measurement conditions rather than an increase in primary particle size. After 48 h, LC_50_ decreased from 322 to 227 mg L^–1^ and LC_90_ from 701 to 420 mg L^–1^. These findings indicate that reaction with Zn^2+^ altered the physicochemical characteristics of rutin and was associated with moderately improved larvicidal activity under the tested laboratory conditions.

## Graphical abstract

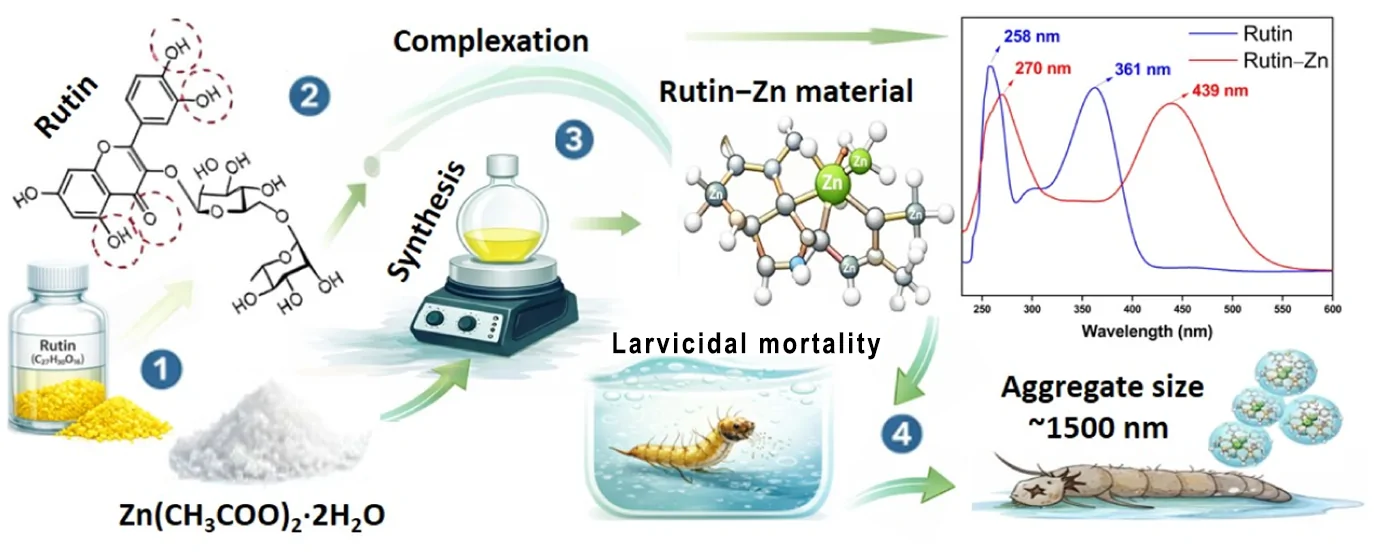

## 1 Introduction

Dengue and other arboviral diseases transmitted by *Aedes aegypti* remain major public-health concerns. Recent surveillance data indicate an unprecedented increase in dengue transmission during 2024, while the expansion of *Aedes*-borne diseases has been associated with urbanization, climate variability, human mobility, and the adaptability of domestic mosquito vectors.^1–3^ Larval control remains important because it targets mosquitoes in aquatic habitats before adult emergence. However, conventional larvicides face limitations related to insecticide resistance, repeated application, environmental concerns, and variable field performance.^4–6^ These challenges have encouraged the investigation of alternative materials with measurable larvicidal activity and physicochemical properties that can be systematically characterized.

Natural products have received renewed attention in vector-control research, including plant extracts, essential oils, nanoemulsions, metal and metal-oxide nanoparticles, and hybrid bioinspired systems.^7–12^ In many studies, however, biological activity is reported without sufficient characterization of the tested material. Larvicidal performance may be influenced by molecular electronic properties,^13^ aggregation state,^14^ aqueous dispersibility,^15^ colloidal stability,^10^ and contact with or ingestion by larvae.^9^ Physicochemical characterization is therefore important for interpreting biological results, although correlations between material properties and mortality should not be treated as proof of a biological mechanism.^11,16^

Flavonoids are polyoxygenated natural compounds capable of hydrogen bonding and interaction with metal ions. Their conjugated aromatic structures are sensitive to protonation, coordination, solvent, and local chemical environment, making them useful for investigating metal-induced changes in electronic and aggregation behavior.^17–19^ Rutin, or quercetin-3-O-rutinoside, is a widely distributed quercetin glycoside^20^ with recognized biological relevance and several potential oxygen-donor groups.^21^ Its practical behavior is nevertheless affected by limited aqueous availability and changes in molecular organization associated with pH,^22^ supramolecular environment,^23^ and formulation conditions.^24^ Previous studies have shown that flavonoids can self-associate, exhibit spectroscopic and structural changes after interaction with metals, and display modified biological properties.^14,25–27^

Metal complexation or association can modify flavonoid behavior through redistribution of electron density,^18^ changes in donor-group acidity and binding equilibria,^28^ variations in preferred interaction sites and conformation,^29^ altered apparent solubility,^30^ and changes in intermolecular organization.^14^ Studies involving quercetin, morin, chrysin, luteolin, and iso-orientin have reported metal-associated changes in antioxidant, antimicrobial, antithrombotic, and other biological responses, accompanied by spectroscopic evidence of ligand–metal interactions.^31–33^ Zn^2+^ is relevant because it is a biologically important, redox-inactive d^1 0^ ion that can interact with oxygen-donor ligands without introducing the redox behavior associated with many transition metals. Flavonoid–zinc interactions may affect ligand physicochemical properties and zinc-dependent biochemical systems.^30,31^ These findings support investigation of rutin–Zn materials, but effects or structures reported for other flavonoids cannot be transferred directly to rutin.

Several materials have been investigated against *A. aegypti*, including nanoemulsions, silver-based materials, metal oxides, chitosan-associated systems, and plant-mediated nanoparticles.^8,34,35^ By comparison, Zn–flavonoid studies have focused mainly on antioxidant,^17,19^ antimicrobial,^36^ anticancer,^36^ antidiabetic, or thrombo-regulatory applications, not on mosquito larvicides.^37^ Rutin differs structurally from quercetin because glycosylation blocks the 3-hydroxyl position and may alter metal-binding and aggregation behavior. Studies of flavonoid systems demonstrate that small structural differences can affect interaction-site preferences, crystal packing, and intermolecular association.^38–40^ Despite previous studies on metal–flavonoid systems, the larvicidal activity of a physicochemically characterized rutin–Zn material against *A. aegypti* remains insufficiently investigated. The present work focuses on combining complementary spectroscopic, diffraction, and DLS measurements with concentration–mortality assays to directly compare the rutin–Zn material with free rutin. This approach provides preliminary evidence that reaction with Zn^2+^ modifies the electronic and aggregation behavior of rutin and is associated with lower LC_50_ and LC_90_ values under controlled laboratory conditions.

In this study, a rutin–Zn material was prepared from rutin and zinc acetate at a nominal 1:1 precursor molar ratio. Its physicochemical characteristics were evaluated using UV–Vis and FTIR spectroscopy, solution NMR, powder X-ray diffraction, and dynamic light scattering. Larvicidal activity against fourth-instar *A. aegypti* larvae was compared with that of free rutin using concentration–mortality assays after 24 and 48 h. The study examined whether reaction with Zn^2+^ produces measurable spectroscopic and aggregation changes and whether the isolated material exhibits a different larvicidal response from free rutin under laboratory conditions. Because zinc content, ligand-to-metal stoichiometry, residual unbound zinc, larval uptake, tissue damage, and biochemical responses were not directly determined, the work does not assign a discrete molecular structure or claim a demonstrated toxicity mechanism. Instead, it provides preliminary physicochemical and biological evidence and identifies questions requiring quantitative metal analysis, zinc and physical-mixture controls, and targeted mechanistic studies.

## 2 Materials and Methods

### 2.1 Preparation of the Rutin–Zn Material

In this study, a rutin–Zn material was prepared according to ref 41, with minor modifications. A 0.4 mol·L^–1^ methanolic solution of zinc acetate dihydrate, Zn­(CH_3_COO)_2_·2H_2_O (Synth, Diadema, Brazil; 99%; 5.0 mL, 2.0 mmol), was added dropwise to a 0.1 mol·L^–1^ methanolic solution of rutin (Sigma-Aldrich, St. Louis, MO, USA; 99%; 20.0 mL, 2.0 mmol), corresponding to a nominal 1:1 Zn:rutin precursor molar ratio. The mixture was stirred at 300 rpm for 24 h at 40 ± 3 °C. The resulting solid was isolated by vacuum filtration, washed with methanol (3 × 2 mL), and dried at room temperature.

### 2.2 Characterization

UV–Vis absorption spectra of rutin and the rutin–Zn material were recorded on a Shimadzu UV-2550 spectrophotometer (Shimadzu Corporation, Kyoto, Japan) over the 190–1100 nm range. Samples were prepared in DMSO at 2 mmol L^–1^ and measured at room temperature (28 °C) in quartz cuvettes with a 1.0 cm path length. FTIR spectra were obtained using a Shimadzu IR Affinity spectrometer (Shimadzu Corporation, Kyoto, Japan) in the 4000–400 cm^–1^ region using the KBr pellet method. Prior to analysis, the samples were dried under vacuum for 4 h. Pellets were prepared by homogenizing each sample with spectroscopic-grade KBr, followed by grinding and compression into thin translucent discs. XRD patterns were collected on a D2 PHASER diffractometer (Bruker AXS SE, Karlsruhe, Germany) operated in continuous PSD fast mode with Cu Kα radiation (λ = 0.15406 nm) at 30 kV and 10 mA. Diffraction data were acquired over 2θ = 5–60° at a scan rate of 3° min^–1^. The diffraction patterns were analyzed using X’Pert HighScore Plus 2.2b software (PANalytical B.V., Almelo, The Netherlands). ^1^H and ^13^C NMR spectra were recorded in DMSO-*d*_6_ at 26 °C on a Varian/Agilent VNMRS spectrometer equipped with a 5 mm liquid-state probe, using the standard s2pul pulse sequence. ^1^H NMR data were acquired at 399 MHz with a spectral width of 6410 Hz, acquisition time of 5.1 s, and 128 scans, whereas ^13^C NMR data were acquired at 100 MHz with broadband ^1^H decoupling, a spectral width of 25,000 Hz, acquisition time of 1.3 s, and 24,000 scans. Hydrodynamic diameter and polydispersity index (PDI) were determined by dynamic light scattering using a Zetasizer Nano ZS (Malvern Instruments Ltd., Malvern, UK) equipped with a 10 mW He–Ne laser operating at 632.8 nm and using noninvasive backscatter detection at 173°. Samples were dispersed in deionized water containing 1% (v/v) DMSO, matching the solvent composition employed in the larvicidal assays, and homogenized by vortex agitation. Measurements were performed 24 h after sample preparation using three technical replicates. Hydrodynamic size distributions were reported on an intensity-weighted basis, and the results were expressed as mean ± standard deviation. The DLS values represent the effective dimensions of solvated scattering entities present in the dispersion and should not be interpreted as primary particle sizes or as direct measurements of the solid-state structural dimensions of the isolated material.

### 2.3 Biological Assay

*Aedes aegypti* larvae were obtained from the Arthropoda Laboratory of the Federal University of Amapá (Macapá, Brazil). Bioassays were conducted under controlled conditions using fourth-instar larvae maintained at 25 ± 2 °C, 75 ± 5% relative humidity, and a 12 h light/12 h dark photoperiod. Free rutin and the rutin–Zn material were initially dissolved in DMSO and subsequently diluted with distilled water to obtain the tested concentrations and a final DMSO concentration of 1% (v/v). A solvent control containing distilled water and 1% (v/v) DMSO was evaluated in parallel.^42^ Each treatment and control consisted of five replicates containing 10 fourth-instar larvae each. Larval mortality was recorded after 24 and 48 h of exposure.

### 2.4 Data Analysis

Prior to statistical analysis, mortality rates were corrected for control mortality using Abbott’s formula. The concentrations required to induce 50% (LC_50_) and 90% (LC_90_) larval mortality, along with their respective confidence intervals, were determined for the 24 and 48 h exposure periods using probit analysis. Statistical significance was defined at *p* ≤ 0.05.

## 3 Results and Discussion

### 3.1 Physicochemical Characterization

The UV–Vis spectra of free rutin and the rutin–Zn material are presented in Figure 1. Free rutin exhibits the characteristic two-band absorption profile of flavonols, with Band II centered at 258 nm and Band Icentered at 361 nm. These two transitions arise from distinct but electronically connected regions of the flavonoid scaffold. Band II is generally associated with the benzoyl-type chromophore of the A/C-ring system, whereas Band Iis attributed to the cinnamoyl-type chromophore involving the B ring and the conjugated C2=C3/C4=O system.^43,44^ Because Band Ireflects the more extended π-conjugated portion of the molecule, it is especially sensitive to changes in electron delocalization, protonation state, hydrogen bonding, solvent environment, and possible interaction with metal ions at oxygenated donor sites. Band II is usually less responsive to such perturbations, but it remains useful for confirming that the aromatic flavonol framework is preserved. The absorption maxima observed for free rutin are consistent with compiled flavonoid spectroscopic data and previous studies reporting bands near 257–258 and 356–358 nm for free rutin.^25,27^ This agreement indicates that the spectrum recorded under the present conditions is coherent with the expected electronic structure of quercetin-3-O-rutinoside. It also provides a reliable baseline for evaluating the spectral changes observed after reaction with Zn^2+^. Thus, any subsequent displacement of Band Ior Band II can be interpreted in relation to the intrinsic electronic behavior of rutin rather than as an unrelated experimental artifact. Accordingly, the free-rutin spectrum supports the validity of the UV–Vis comparison and establishes an appropriate reference for discussing the electronic effects associated with formation of the rutin–Zn material.

**Figure 1:**
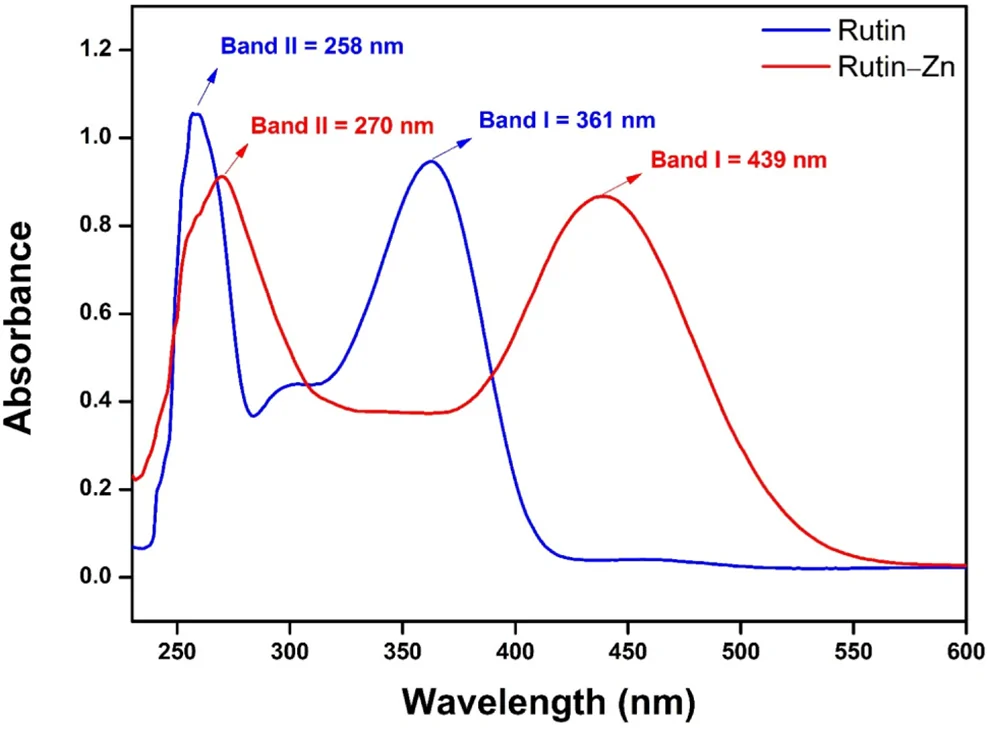
UV–Vis absorption spectra of free rutin and the rutin–Zn material in DMSO.

After reaction with Zn^2+^, Band II shifts from 258 to 270 nm, whereas Band I undergoes a substantially larger bathochromic shift from 361 to 439 nm. The 78 nm displacement of Band Iis the principal spectral change and indicates a marked perturbation of the conjugated electronic system of rutin. Because Band Iis particularly sensitive to the cinnamoyl-type chromophore, this shift is consistent with a decrease in the energy of the corresponding electronic transition and redistribution of electron density within the flavonoid framework. Similar spectral behavior has been reported for other Zn–flavonoid systems, including zinc–quercetin and zinc–morin materials, in which Zn^2+^ association produces a relatively modest change in Band II and a more pronounced bathochromic displacement of Band I.^32,33^ More broadly, shifts in the principal UV–Vis absorption bands are commonly employed as evidence of metal–flavonoid interactions.^18,28,32^ Nevertheless, the UV–Vis results alone cannot establish a unique coordination site, molecular geometry, or rutin:Zn stoichiometry. Metal binding in flavonoids may involve oxygen-donor regions containing carbonyl and hydroxyl groups, including the 4-carbonyl/5-hydroxyl region or catechol-type sites, depending on the ligand structure, protonation state, solvent, and metal ion.^28^ This distinction is particularly relevant for rutin because glycosylation at the 3-hydroxyl position prevents the conventional 3-OH/4-keto coordination mode available to quercetin. Therefore, the observed bathochromic shifts are most appropriately interpreted as evidence that reaction with Zn^2+^ generates a modified electronic environment involving oxygenated regions of rutin, rather than as proof of a specific chelation mode. Taken together with the complementary FTIR and NMR results, the UV–Vis data support an interaction between Zn^2+^ and rutin but do not independently define the composition or structure of the resulting rutin–Zn material.

The FTIR results provide complementary evidence of changes in the local bonding environment after reaction with Zn^2+^. As shown in Figure 2 and summarized in Table 1, the broad ν­(O–H) band shifts from 3391 cm^–1^ for free rutin to 3163 cm^–1^ for the rutin–Zn material. This displacement indicates substantial modification of the hydroxyl environments and may arise from interaction between Zn^2+^ and oxygen-donor groups, changes in hydrogen bonding, differences in hydration, or a combination of these effects.^19,45^ Therefore, the O–H shift supports perturbation of oxygenated regions of rutin but does not independently demonstrate hydroxyl deprotonation or identify a specific coordination site. The conjugated carbonyl band shows only a small shift from 1657 to 1653 cm^–1^, indicating that its local electronic environment is affected without providing conclusive evidence of direct carbonyl coordination.^32,33,36^ A more pronounced change occurs in the aromatic and conjugated region, where the band assigned primarily to ν­(C=C) shifts from 1601 to 1560 cm^–1^ and the feature at 1555 cm^–1^ is no longer resolved. These changes are consistent with modification of vibrational coupling and electron distribution within the flavonoid framework following interaction with Zn^2+^.^18,32,46^ Nevertheless, variations in intermolecular hydrogen bonding, hydration, and solid-state organization may also contribute to the observed band positions and profiles. Accordingly, the combined UV–Vis and FTIR results support the formation of a modified electronic and bonding environment in the rutin–Zn material, but they do not establish a unique coordination geometry, binding site, or stoichiometry.

**Figure 2:**
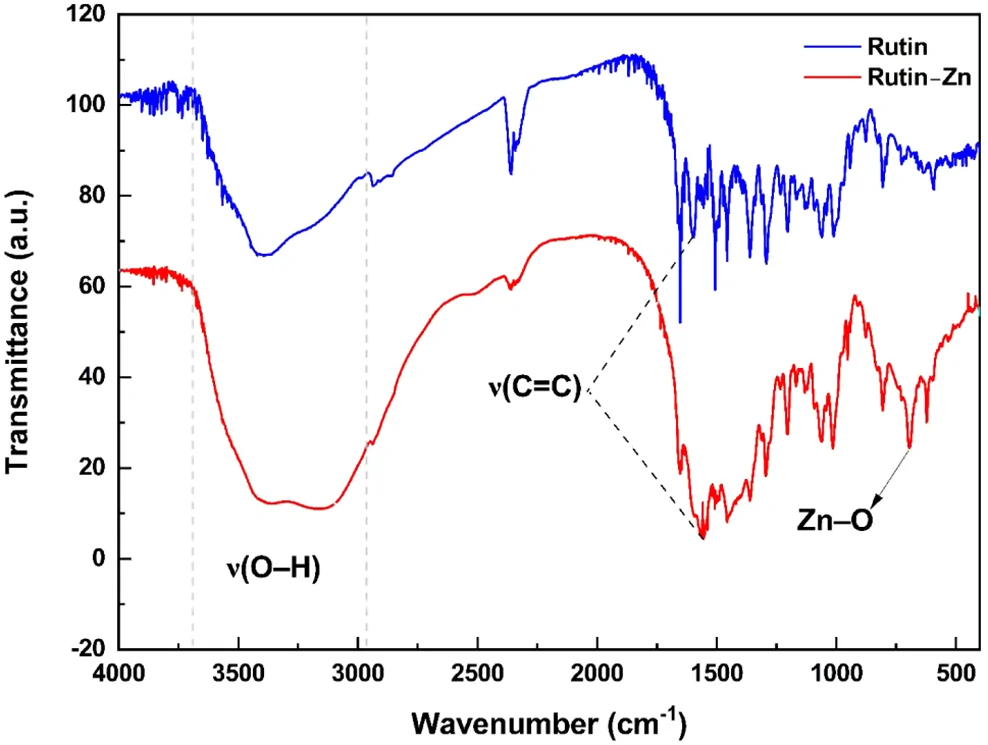
FTIR spectra of free rutin and the rutin–Zn material in the 4000–400 cm^–1^ region.

**Table 1:** Observed FTIR Bands for Free Rutin and the Rutin–Zn Material^a^

Assignment	Free rutin (cm^–1^)	Rutin–Zn (cm^–1^)	Spectral change	Refs
ν(O–H)*, broad stretching	3391	3163	Strong shift to lower wavenumber	^19^,^45^
ν(C–H), weak stretching	2932	-	Disappears/strongly attenuated	^47–49^
ν(C=O), conjugated carbonyl	1657	1653	Small shift	^32^,^33,36^
ν(C=C)*, aromatic/conjugated	1601	1560	Pronounced shift to lower wavenumber	^18^,^32,46^
Aromatic skeletal vibration	1555	-	Absent or merged	^18^,^32,46^
Aromatic skeletal vibration	1501	1499	Minor shift	^32^,^49,50^
Aromatic skeletal vibration/ring deformation	1458	1451	Minor shift	^32^,^38,50^
ν(C–OH)/phenolic vibration	1362	1362	No significant shift	^46^,^51^
ν(C–O–C)	1290	1290	No significant shift	^18^,^52,53^
ν(C–O)	1206	1206	No significant shift	^18^,^38^
ν(C–O)	1161	1163	Minor shift	^18^,^38,52^
ν(C–O)	1125	1126	Minor shift	^38^,^52^
ν(C–O)	1063	1065	Minor shift	^18^,^38,52^
Ring/glycosidic vibration	1005	1015	Shift to higher wavenumber	^26^,^54,55^
Out-of-plane aromatic deformation	880	-	Absent	^18^,^56,57^
Out-of-plane aromatic deformation	800	804	Minor shift	^18^,^56,57^
Low-frequency band tentatively associated with a Zn–O vibration*	-	694	New band appears only in rutin–Zn material	^58^
Out-of-plane deformation	719	-	Absent	^38^,^57,59^
Low-frequency deformation	633	623	Shift to lower wavenumber	^38^,^57,60^

a Bands marked with an asterisk (*) highlight the main spectral differences between the two samples and indicate the most relevant changes observed after reaction with Zn^2+^.

Equally important, the FTIR fingerprint region remains broadly similar after reaction with Zn^2+^. As shown in Table 1, the ν­(C–O–C) band at 1290 cm^–1^ and the ν­(C–O) band at 1206 cm^–1^ remain essentially unchanged. The bands at 1161, 1125, and 1063 cm^–1^ exhibit only small displacements, while the ring/glycosidic mode shifts from 1005 to 1015 cm^–1^.^18,26,38,52,54,55^ These observations suggest that the ether and glycosidic framework of rutin is largely retained in the rutin–Zn material. However, FTIR alone cannot exclude limited chemical or conformational changes in the rutinoside moiety. A new band is observed at 694 cm^–1^ in the rutin–Zn sample. This feature may be consistent with a Zn–O-related vibration, as low-frequency metal–oxygen modes have been reported for other metal–flavonoid systems.^18,32,46^ Nevertheless, this assignment should be considered tentative because ligand deformation, out-of-plane vibrations, residual zinc acetate, and differences in the solid-state environment may also contribute in this spectral region. The disappearance of the bands at 880 and 719 cm^–1^, together with the small displacements from 800 to 804 cm^–1^ and from 633 to 623 cm^–1^, indicates modification of the vibrational environment and possibly the molecular packing of rutin.^38,57,59,60^ Overall, the FTIR data support preservation of the principal rutin framework accompanied by changes in its hydroxyl-associated and conjugated environments. When considered alongside the UV–Vis and NMR results, these changes are consistent with an interaction between Zn^2+^ and rutin, but they do not independently establish a unique coordination site, geometry, stoichiometry, or discrete molecular structure.

The powder XRD pattern of the isolated reaction product is shown in Figure 3. The prominent Bragg reflections at 2θ ≈ 12.3, 14.4–16.7, 22.0, and 26.3–26.8° are predominantly consistent with crystalline rutin (C_27_H_30_O_16_, PDF#00-005-0444) and zinc acetate dihydrate [Zn­(C_2_H_3_O_2_)_2_·2H_2_O, PDF#00-009-0676]. These reflections indicate the persistence of residual crystalline precursors in the isolated product. No distinct set of Bragg reflections attributable to a new crystalline rutin–Zn phase was identified. The diffuse background additionally indicates the presence of an X-ray-amorphous fraction. When considered together with the spectroscopic results, it is plausible that the amorphous fraction contains Zn-associated rutin species; however, this assignment cannot be established by powder XRD. The technique also cannot determine the chemical identity, composition, stoichiometry, or local coordination geometry of the amorphous component. The diffraction results therefore do not demonstrate the formation of a phase-pure or crystalline rutin–Zn material. Instead, the sample is best described as a heterogeneous isolated product containing an X-ray-amorphous fraction and residual crystalline rutin and zinc acetate dihydrate. This interpretation distinguishes the spectroscopic evidence of Zn^2+^–rutin interaction from the long-range structural information obtained by powder XRD.

**Figure 3:**
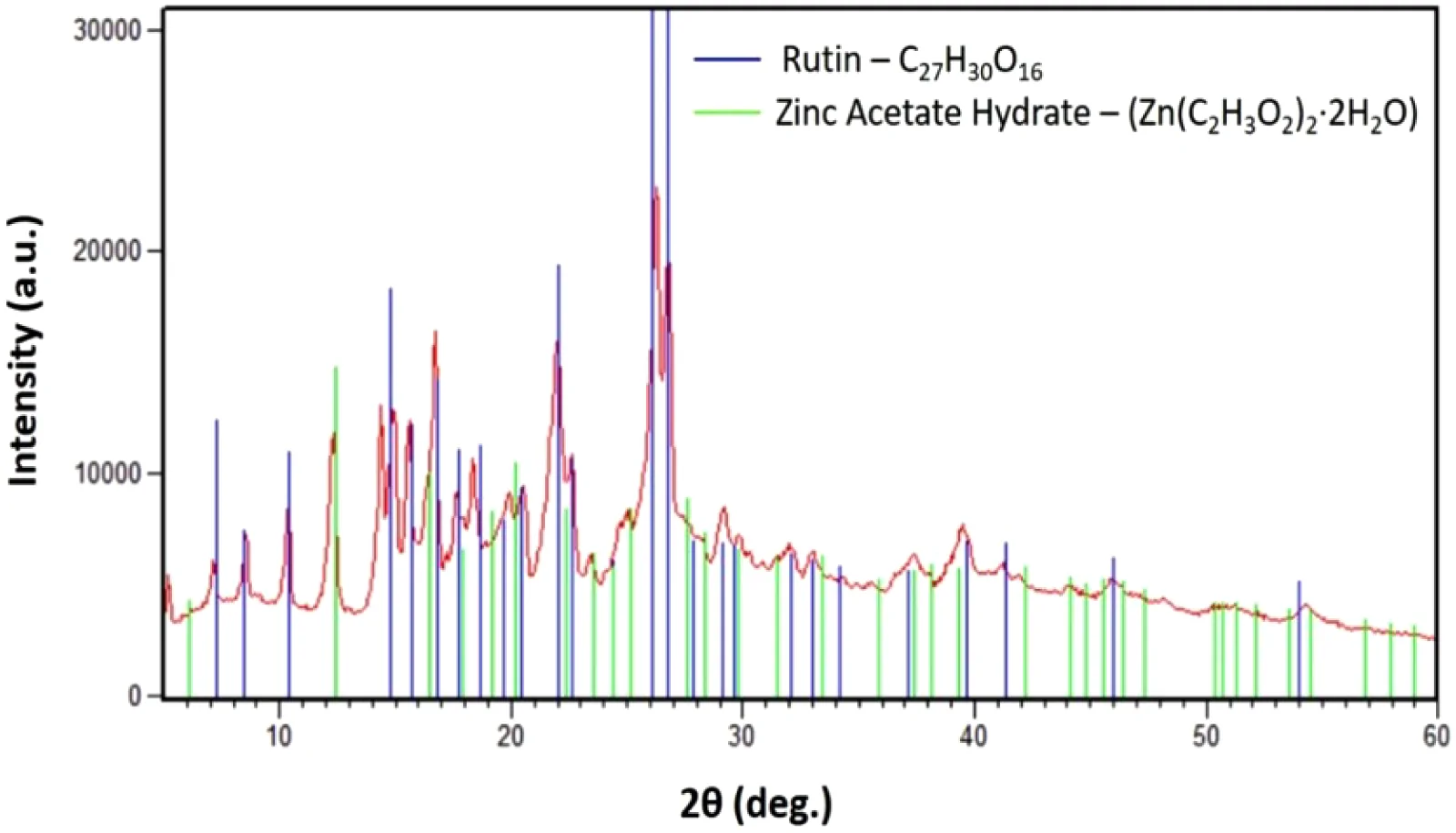
Powder XRD pattern of the isolated reaction product compared with the reference reflections of crystalline rutin and zinc acetate dihydrate.

The NMR results provide complementary information on the local chemical environment of rutin after reaction with Zn^2+^. Whereas powder XRD evaluates long-range structural order in the isolated solid, solution NMR detects changes in the electronic environments of individual ligand nuclei. Because the spectra were recorded in DMSO-*d*_6_, however, the observed species may be influenced by solvation, ligand exchange, or partial dissociation. The results should therefore be interpreted as evidence of Zn^2+^-induced perturbation rather than definitive proof of a unique coordination geometry. Figure 4 presents the ^1^H and ^13^C NMR spectra of the rutin–Zn sample, while Table 2 compares its resonances with an external experimental data set for pure rutin recorded at 400 MHz in DMSO-*d*_6_.^61^ The assignments in Table 2 are tentative and were obtained by comparing corresponding spectral regions. In the ^1^H spectrum, signals tentatively associated with 5-OH, 7-OH, 3′-OH, and 4′-OH were not resolved, indicating alteration of the phenolic environments. Nevertheless, their disappearance cannot independently establish deprotonation or coordination because proton exchange, residual water, line broadening, concentration, and hydrogen bonding may also suppress these resonances. Changes were also observed in the aromatic region: H-8 shifted from approximately 6.40 to 6.30 ppm, H-5′ from 6.86 to 6.70 ppm, and H-2′/H-6′ from approximately 7.55–7.56 to 7.53 ppm. These differences indicate electronic perturbation of the flavonoid core and B ring. Conversely, the CH_3_-6′′′ resonance of rhamnose remained essentially unchanged at approximately 0.99 ppm, suggesting preservation of the terminal rhamnose environment. The apparent displacement of H-1′′′ from approximately 5.12 to 4.39 ppm must be interpreted cautiously because it could reflect conformational reorganization, altered hydrogen bonding, or spectral overlap rather than direct coordination to the carbohydrate unit.

**Figure 4:**
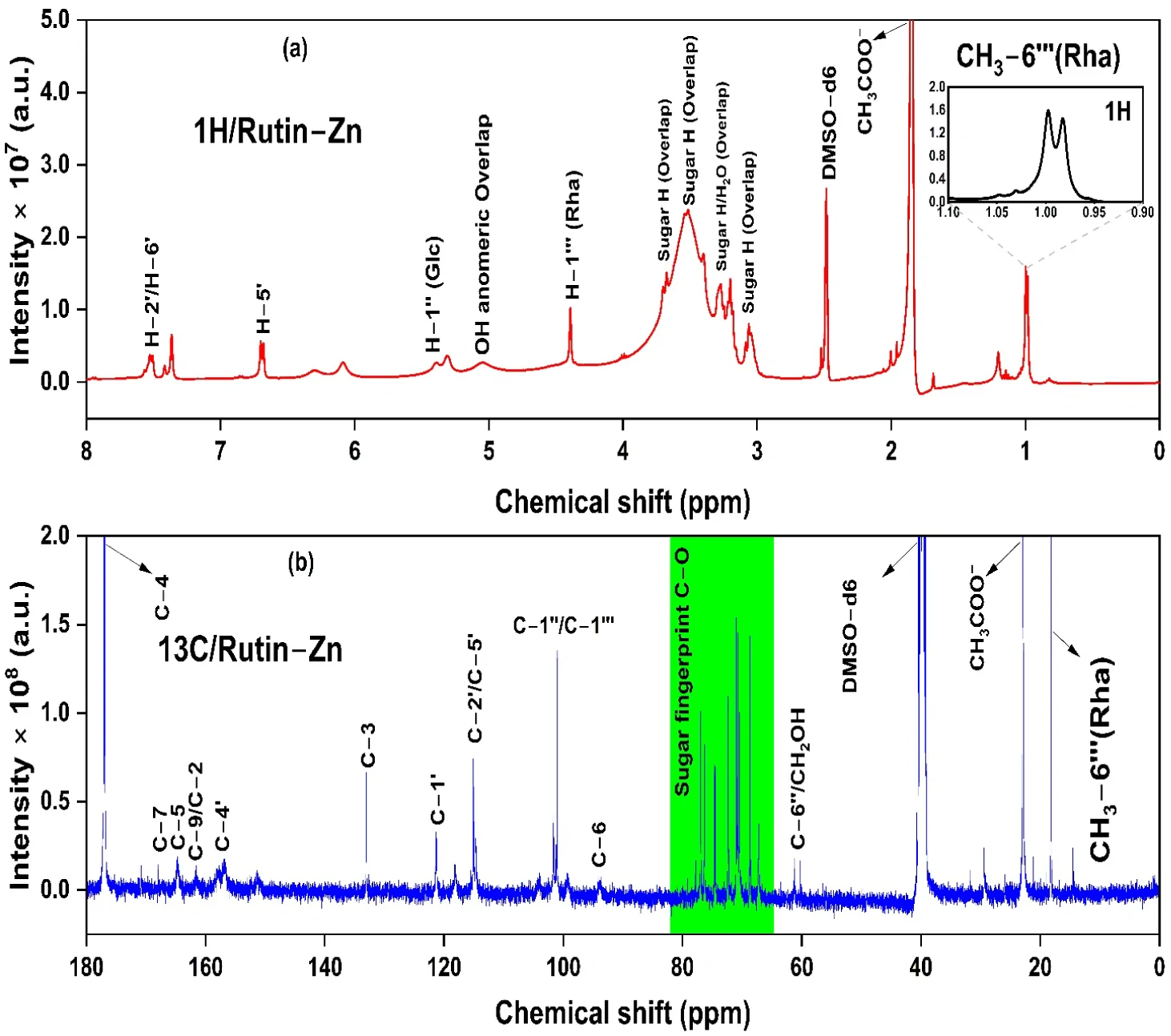
(a) ^1^H and (b) ^13^C NMR spectra of the rutin–Zn material in DMSO-*d*_6_. The main resonances of the flavonoid nucleus and sugar residues are indicated, with inset expansion of the CH_3_-6‴ resonance of the rhamnose unit in the ^1^H spectrum. The green-shaded region in the ^13^C spectrum denotes the congested carbohydrate fingerprint region.

**Table 2:** Comparison of the ^1^H and ^13^C NMR Chemical Shifts of Free Rutin and the Rutin–Zn Sample^a^

Signal	Free rutin δ (ppm)	Rutin–Znδ (ppm)	Δδ (ppm)*	Interpretation
5-OH (^1^H)	12.62	Not observed	–	Disappearance indicates a perturbed phenolic environment; not diagnostic of coordination/deprotonation
7-OH (^1^H)	10.86	Not observed	–	Altered hydrogen-bonding/electronic environment
4′-OH (^1^H)	9.71	Not observed	–	Phenolic environment perturbed after reaction with Zn^2+^
3′-OH (^1^H)	9.21	Not observed	–	Phenolic environment perturbed after reaction with Zn^2+^
H-8 (^1^H)	6.40	6.30	–0.1	Moderate shielding
H-6 (^1^H)	6.21	6.20	–0.01	Essentially preserved
H-5′ (^1^H)	6.86	6.70	–0.16	Noticeable shift
H-2″/H-6″ (^1^H)	7.55–7.56	7.53	–0.02 to – 0.03	Small aromatic perturbation
H-1″ (Glc, ^1^H)	5.35	5.31	–0.04	Sugar moiety weakly affected
H-1′′′ (Rha, ^1^H)	5.12	4.39	–0.73	Strong perturbation in glycosidic region
CH_3_-6′′′ (Rha, ^1^H)	1.00	0.99	–0.01	Rhamnose methyl retained
C-2 (^13^C)	157.3	156.99	–0.31	Minor change
C-3 (^13^C)	134.1	133.05	–1.05	Moderate perturbation
C-4 (^13^C)	178.2	176.97	–1.23	Carbonyl environment perturbed
C-5 (^13^C)	157.5	161.58	+4.08	Strong perturbation near a plausible Zn^2+^-interaction region
C-6 (^13^C)	99.5	99.35	–0.15	Essentially preserved
C-7 (^13^C)	164.9	164.69	–0.21	Small change
C-8 (^13^C)	94.5	Not assigned	–	Not clearly resolved
C-9 (^13^C)	162.1	Not unambiguously assigned	–	Overlapped/partially unresolved
C-10 (^13^C)	104.8	104.33	–0.47	Minor change
C-1′ (^13^C)	122.5	121.30	–1.20	Moderate perturbation
C-2′ (^13^C)	116.1	115.06	–1.04	Moderate perturbation
C-3′ (^13^C)	145.6	Not assigned	–	Likely overlapped in oxygenated aromatic region
C-4′ (^13^C)	149.3	151.40	+2.10	Relevant electronic perturbation
C-5′ (^13^C)	117.1	115.06	–2.04	Moderate perturbation
C-6′ (^13^C)	122.0	Not assigned	–	Not clearly resolved
C-1″ (Glc, ^13^C)	101.6	101.05	–0.55	Sugar anomeric carbon preserved
C-2″ (Glc, ^13^C)	74.9	74.59	–0.31	Minor change

a The reference values for free rutin were obtained from a publicly available experimental NMR dataset recorded at 400 MHz in DMSO-d_6_.^61^ Chemical-shift differences were calculated as Δδ = δ­(rutin–Zn) – δ­(free rutin). Approximate assignments were made by comparison of the corresponding spectral regions. Blank fields (−) indicate that no direct and unambiguous assignment was possible for the rutin–Zn sample.

The ^13^C NMR spectrum provides further evidence of electronic perturbation in the flavonoid region. Among the tentatively assigned resonances, C-5 exhibits the largest displacement, changing from approximately 157.5 ppm in the external free-rutin spectrum to 161.58 ppm in the rutin–Zn sample. Changes are also observed for the C-4 carbonyl resonance and C-3, collectively supporting perturbation near the 5-hydroxyl/4-carbonyl region. Because the 3-hydroxyl group of quercetin is glycosylated in rutin, the classical 3-OH/4-keto chelation mode is unavailable. Changes tentatively assigned to C-4′, C-2′, and C-5′ further indicate that the catechol-containing B ring is electronically affected. These differences could result from participation of the 3′,4′-dihydroxyl region, redistribution of electron density through the conjugated flavonoid framework, or altered hydrogen bonding; the available one-dimensional spectra cannot distinguish conclusively among these possibilities. Thus, the combined perturbation of multiple ^1^H and ^13^C environments supports an interaction between Zn^2+^ and the oxygenated regions of rutin. Nevertheless, the data do not exclude coexistence with residual or physically mixed components, nor do they establish a unique coordination site or demonstrate the presence of only one rutin–Zn species in DMSO-*d*_6_. The 5-OH/4-keto region should therefore be described as a chemically plausible coordination domain that is consistent with, but not uniquely demonstrated by, the available NMR evidence.

The DLS profiles in Figure 5 indicate that reaction with Zn^2+^ alters the aggregation behavior of rutin under the measurement conditions. Free rutin exhibited an intensity-weighted average hydrodynamic diameter of 226 ± 3 nm and a polydispersity index (PDI) of 0.28 ± 0.02. Its bimodal intensity distribution, comprising a dominant submicrometric population and a weaker contribution in the micrometric range, indicates the coexistence of aggregates with different apparent sizes. The rutin–Zn material exhibited a substantially larger average hydrodynamic diameter of 1501 ± 19 nm and a higher PDI of 0.44 ± 0.11. Although its intensity distribution appeared predominantly unimodal, the increased PDI demonstrates that the sample was more polydisperse and therefore should not be described as more homogeneous than free rutin. The relatively large uncertainty associated with its PDI also indicates variability among replicate measurements. DLS measures the hydrodynamic diameter of solvated scattering entities rather than the primary particle size or dry-state structural dimensions of the isolated material. The measured value includes contributions from the scattering entity, its associated solvent layer, and any aggregation occurring in the dispersion. DLS does not independently determine aggregate morphology, internal structure, chemical composition, crystallinity, or the dimensions of individual primary particles. Moreover, because scattering intensity is strongly weighted toward larger particles, a comparatively small number of large aggregates may substantially affect the measured distribution.^62^ Therefore, the hydrodynamic diameter of 1501 ± 19 nm should not be interpreted as the primary particle size, molecular dimensions, or solid-state structural dimensions of a discrete rutin–Zn species, nor as evidence of improved dispersion or structural uniformity. Instead, the simultaneous increase in hydrodynamic diameter and PDI indicates that reaction with Zn^2+^ modifies the association state of rutin and promotes the formation of larger, compositionally or dimensionally heterogeneous solvated aggregates under the DMSO–water conditions employed. This interpretation is compatible with the environment-dependent self-association of flavonoids^14^ and reports that metal interactions can modify the intermolecular organization of flavonoid-based materials.^18^ Accordingly, the structural nature of the isolated solid must be considered from the complementary XRD and spectroscopic results, whereas DLS provides information only about its aggregation state in the DMSO–water dispersion.

**Figure 5:**
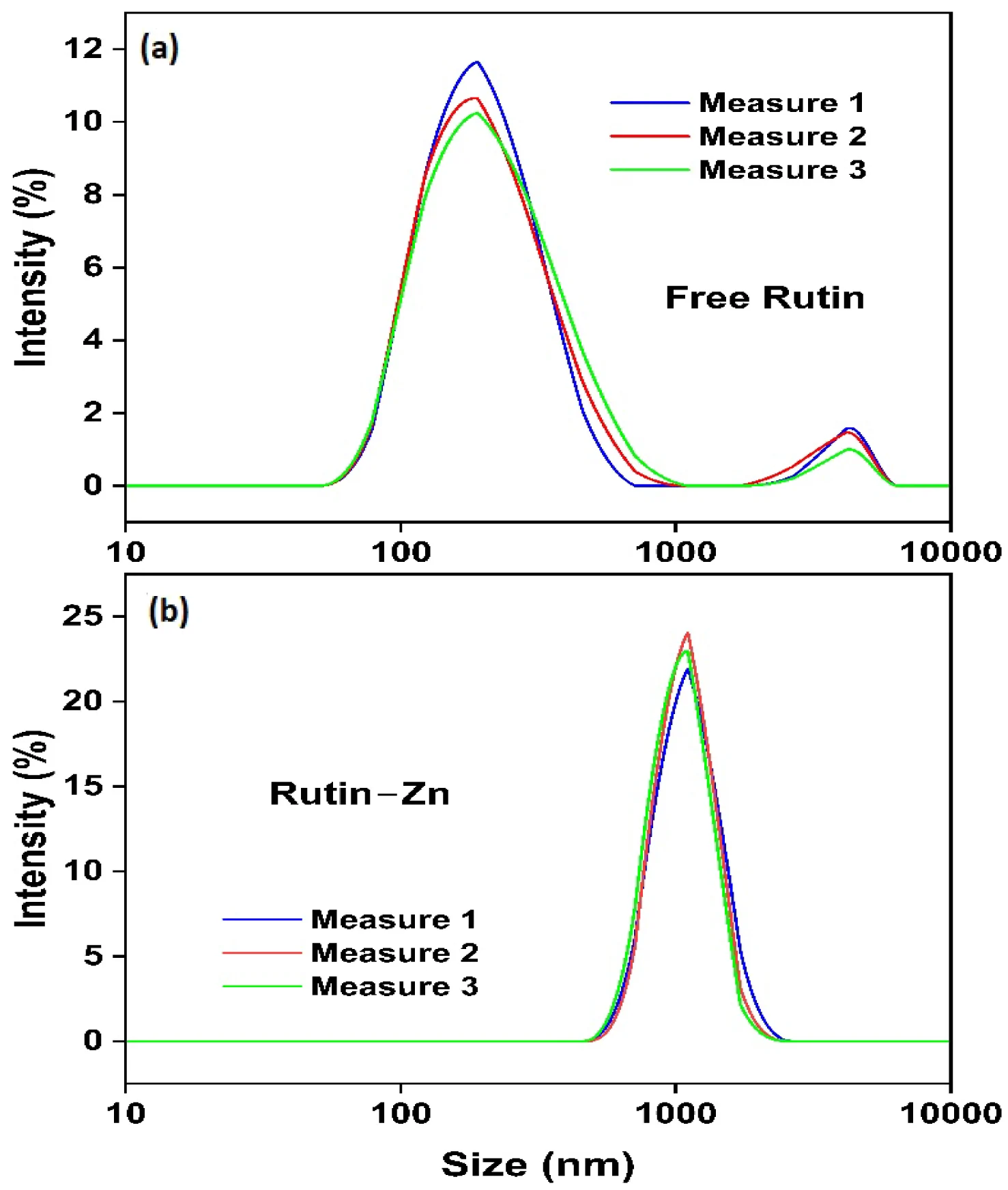
Intensity-weighted DLS hydrodynamic size distributions of (a) free rutin and (b) the rutin–Zn material in deionized water containing 1% (v/v) DMSO.

The combined spectroscopic results support an interaction between Zn^2+^ and rutin, although the available data do not permit unambiguous determination of the stoichiometry, molecular geometry, or formation pathway of the resulting material. Because Zn^2+^ is a redox-inactive d^10^ ion, the observed spectroscopic changes are consistent with Zn^2+^–rutin association involving oxygen-donor regions and are not indicative of oxidative modification of rutin.^18^ Previous studies have shown that Zn^2+^ can interact with O,O-donor regions of flavonoids, with binding preferences depending on ligand structure, protonation state, solvent, and reaction conditions.^28,29^ In rutin, rutinosylation blocks the 3-hydroxyl group and prevents the conventional 3-OH/4-carbonyl coordination mode available to quercetin. The 5-OH/4-carbonyl region therefore represents a plausible Zn^2+^-interaction region, although possible involvement of the 3′,4′-dihydroxyl region cannot be excluded. This interpretation is consistent with the bathochromic shift of UV–Vis Band I, displacement of the O–H stretching band, and NMR perturbations observed near the C-5 and C-4 environments. The new FTIR band at 694 cm^–1^ may also be associated with a Zn–O-related vibration, but this tentative assignment does not establish a particular coordination geometry. Powder XRD indicates that the isolated product is heterogeneous, containing an X-ray-amorphous fraction together with residual crystalline rutin and zinc acetate dihydrate; no distinct crystalline rutin–Zn phase was identified. DLS further revealed larger hydrodynamic entities and increased polydispersity after reaction with Zn^2+^, demonstrating altered aggregation behavior under the measurement conditions. However, the interactions responsible for this behavior cannot be determined from DLS. Hydrogen bonding, aromatic interactions, and solvent-mediated association may contribute, but their respective roles remain unresolved. Accordingly, the product is best described as a heterogeneous isolated material exhibiting Zn^2+^-associated spectroscopic changes and altered aggregation behavior, without assignment of a unique molecular structure, composition, coordination geometry, or assembly pathway.

### 3.2 Larvicidal Performance

The concentration–mortality results indicate that the isolated rutin–Zn material produced lower LC_50_ and LC_90_ estimates than free rutin under the tested laboratory conditions (Figure 6a–d). After 24 h, free rutin exhibited limited activity within the evaluated concentration range. Because mortality did not reach the levels required for reliable estimation and the concentration–response model showed a poor fit (r^2^ = 0.175), the LC_50_ and LC_90_ could not be reliably determined within the tested range of 100–700 mg L^–1^. Extrapolated values were therefore not considered quantitatively meaningful. After 48 h, free rutin produced estimable LC_50_ and LC_90_ values of 322 and 701 mg L^–1^, respectively, with an improved model fit (r^2^ = 0.946). This result indicates a time-dependent concentration–mortality response for free rutin. The rutin–Zn material produced LC_50_ and LC_90_ values of 408 and 683 mg L^–1^, respectively, after 24 h and 227 and 420 mg L^–1^ after 48 h. The corresponding models showed r^2^ values of approximately 0.96 at both exposure times. Thus, the rutin–Zn material produced lower LC_50_ and LC_90_ point estimates than free rutin, particularly after 24 h. At 48 h, the LC_50_ and LC_90_ estimates were approximately 30% and 40% lower, respectively, than those determined for free rutin. These findings indicate a stronger concentration–mortality response for the isolated material than for free rutin under the specific conditions examined. Nevertheless, the absolute activity remained moderate, and the results should not be interpreted as demonstrating practical effectiveness under environmental or field conditions.

**Figure 6:**
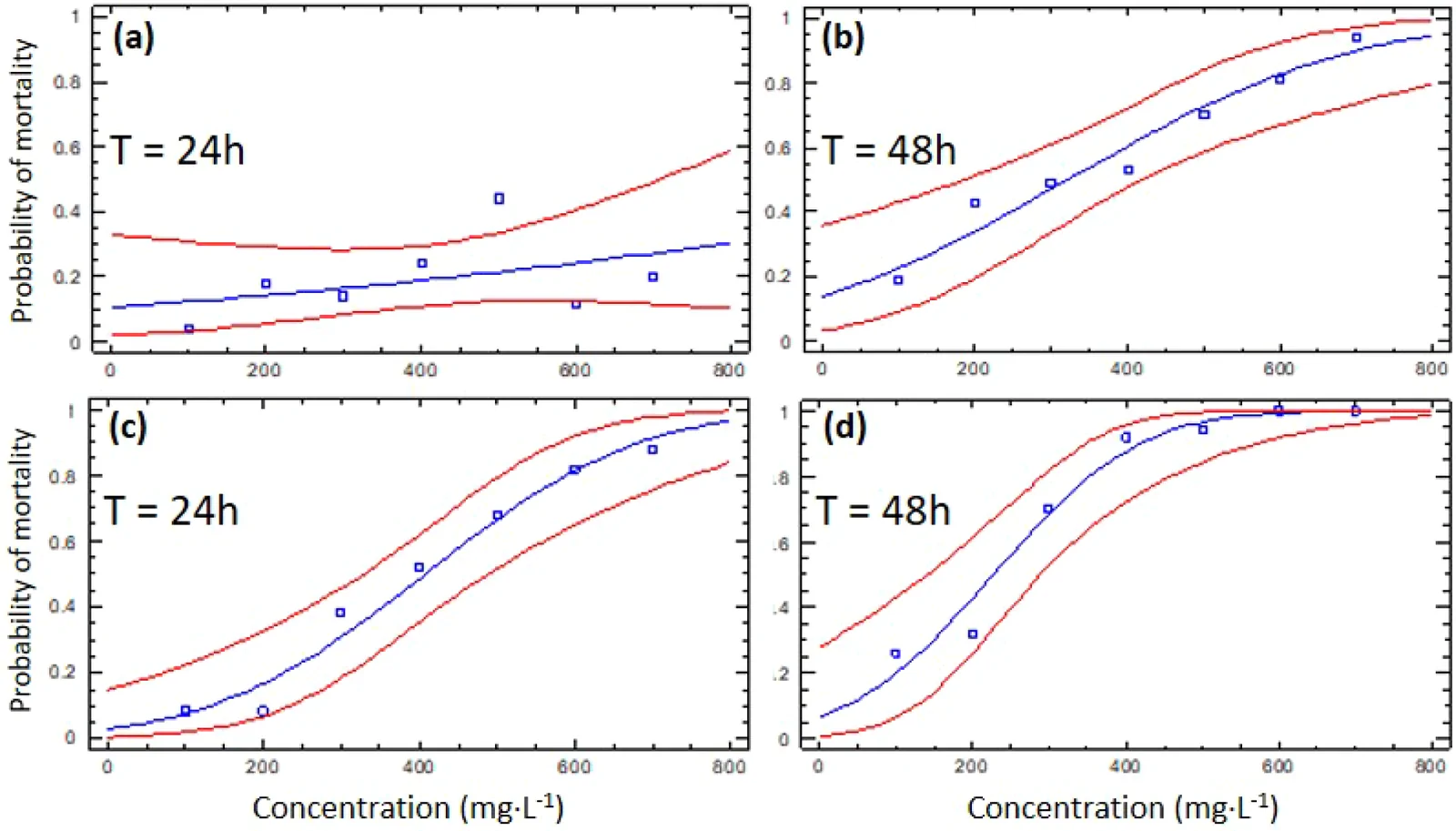
Dose–response curves fitted to the probability of mortality of *Aedes aegypti* larvae, with corresponding 95% confidence limits, after treatment with (a) free rutin for 24 h, (b) free rutin for 48 h, (c) the rutin–Zn material for 24 h, and (d) the rutin–Zn material for 48 h.

The literature systems summarized in Table 3 are included only to provide broad contextual information and not as directly equivalent quantitative benchmarks. Differences in material composition, mosquito strain, larval instar, exposure time, assay protocol, mortality end point, and concentration units prevent rigorous comparison or ranking of larvicidal potency across studies. Therefore, the biological performance of the rutin–Zn material should be evaluated primarily through its direct comparison with free rutin under identical experimental conditions. Its LC_50_ after 48 h was 227 mg L^–1^, compared with 322 mg L^–1^ for free rutin, corresponding to an approximately 30% reduction. The LC_90_ decreased from 701 to 420 mg L^–1^, representing an approximately 40% reduction. These results demonstrate that the isolated rutin–Zn material produced a stronger concentration–mortality response than free rutin under the tested conditions. However, because zinc acetate and rutin–zinc acetate physical-mixture controls were not included, this improvement cannot be attributed specifically to Zn^2+^–rutin association. Residual zinc acetate, released Zn^2+^, or combined exposure to rutin and zinc may also have contributed to the observed activity. Several nanoenabled larvicidal platforms listed in Table 3 report numerically lower LC_50_ values than the rutin–Zn material. However, these values were obtained using different formulations, larval stages, exposure periods, concentration units, and experimental protocols and therefore should not be interpreted as evidence of directly superior potency. These include Ag/AgCl systems,^63^ plant-mediated CeO_2_ nanoparticles,^8^ *Curcuma xanthorrhiza* formulations,^64^ *Ocotea odorifera* nanoemulsions,^65^ and *Cinnamomum zeylanicum* oil nanoemulsions.^66^ Consequently, the present material should not be described as one of the most potent larvicides reported against *Aedes aegypti*. Its relevance is instead limited to the preliminary observation that reaction with Zn^2+^ alters the physicochemical characteristics of rutin and is associated with lower LC_50_ and LC_90_ estimates relative to free rutin. Comparisons among these systems must be treated cautiously because the studies differ in material composition, formulation, mosquito strain, larval stage, exposure period, assay conditions, mortality criteria, and concentration units. Consequently, the reported LC_50_, LC_90_, and mortality values are not directly equivalent across studies. Hydrodynamic diameter alone does not determine larvicidal activity, and direct correlations between particle size and toxicity are not justified by the present results. Smaller systems, including copaiba oleoresin nanoemulsions^7^ and *Pterodon emarginatus* nanodispersions,^67^ have shown greater activity, while materials with larger or broader particle-size distributions, such as β-myrcene–chitosan nanoparticles^9^ and carbon-black aggregates,^68^ may also exhibit biological effects. Nevertheless, these comparisons do not demonstrate that aggregation, dispersibility, interfacial behavior, or larval uptake explains the activity of the rutin–Zn material. Therefore, the comparative analysis supports a cautious conclusion: the isolated rutin–Zn material exhibits moderate larvicidal activity and improves the concentration–mortality response relative to free rutin under the conditions investigated. The relationship between its physicochemical characteristics and biological performance remains hypothetical. Appropriate precursor and physical-mixture controls, zinc-content and release measurements, uptake studies, and mechanistic biological assays will be necessary to determine whether the observed improvement originates from Zn^2+^–rutin association, residual zinc species, altered aggregation, or a combination of these factors.

**Table 3:** Contextual Overview of Larvicidal Systems Reported against *Aedes aegypti*, Including Size-Related Data and Biological Performance, Together with Free Rutin and the Rutin–Zn Material Evaluated in the Present Study^a^

System/material	Target/stage	Reported size-related data and measurement basis	Larvicidal performance reported	Ref
Rutin–Zn material	*Aedes aegypti* larvae	Hydrodynamic diameter = 1501 ± 19 nm, intensity-weighted DLS; aggregated dispersion	LC_50_ = 227 mg·L^–1^ (48 h); LC_90_ = 420 mg·L^–1^	Present work
Free rutin	*Aedes aegypti* larvae	Hydrodynamic diameter = 226 ± 3 nm, intensity-weighted DLS; aggregated populations	LC_50_ = 322 mg·L^–1^ (48 h); LC_90_ = 701 mg·L^–1^	Present work
*Phaseolus vulgaris* ZnO/CuO nanoparticles	*Aedes aegypti*, fourth instar	CuO: 22.6 nm; ZnO: 662 nm	Tested at 6.25–100 μg·L^–1^; ZnO reported superior to CuO	^69^
Plant-mediated CeO_2_ nanoparticles	*Aedes aegypti*, fourth instar	40–60 nm	LC_50_ = 46.28 μg·L^–1^ (24 h); AChE and GST inhibition reported	^8^
Cymene and myrcene nanoemulsions	*Aedes aegypti*, third instar	Cymene NE: 98 nm; Myrcene NE: 118 nm	Significant lethality reported at 50 mg·L^–1^	^10^
β-Myrcene–chitosan nanoparticles	*Aedes aegypti* larvae	30–2800 nm; zeta potential: +15 mV	100% mortality at 238 mg·L^–1^	^9^
Ag/AgCl nanoparticles	*Aedes aegypti* larvae	NR	LC_50_ = 0.133–0.217 μg·L^–1^	^63^
Silk-sericin-capped Ag nanobioinsecticides	*Aedes aegypti*, fourth instar	120 nm (best formulation)	Best formulation: LC_90_ = 478.45 ppm	^35^
Ag nanoparticles	*Aedes aegypti* larvae	NR	LC_50_ = 0.10 ppm	^70^
Copaiba oleoresin nanoemulsion	*Aedes aegypti* larvae	11.5 ± 0.2 to 257.3 ± 4.1 nm	93.3% mortality at 250 ppm after 48 h	^71^
*Pterodon emarginatus* nanoemulsion	*Aedes aegypti* larvae	160–300 nm	LC_50_ = 34.75 ppm (48 h); 100% mortality at 250 ppm	^67^
*Curcuma xanthorrhiza* essential-oil nanoemulsion	*Aedes aegypti* larvae	NR	LC_50_ = 25.94 mg·L^–1^	^64^
*Ocotea odorifera* (sassafras) nanoemulsion	*Aedes aegypti* larvae	<200 nm; PDI < 0.1	LC_50_ = 38 ppm; LC_90_ = 46 ppm	^65^
*Caulerpa serrulata* extract/TiO_2_ nanoparticles	*Aedes aegypti* larvae	Extract: 13.012–275.177 μm; TiO_2_–NPs: 0.012–2000 μm	Extract LC_50_ = 0.16 ppm; TiO_2_–NPs LC_50_ = 0.14 ppm	^72^
*Annona mucosa* ethanolic extract/acetogenins	*Aedes aegypti*, third–fourth instar	NR	Extract LC_50_ = 2.60 μg·L^–1^; isolated compounds 0.43–0.78 μg/mL	^7^
Carbon black nanoparticles (E1800)	*Aedes aegypti*, first instar	Primary particles 25–37 nm; aggregates 140–220 nm	LC_50_ = 0.42 mg·mL^–1^ (susceptible strain) and 0.72 mg/mL (resistant strain)	^68^
*Cinnamomum zeylanicum* oil nanoemulsion	*Aedes aegypti* larvae	NR	Essential oil LC_50_ = 22.01 mg·L^–1^; nanoemulsion LC_50_ = 5.30 mg·L^–1^	^66^

a The compiled studies differ substantially in material composition, mosquito strain, larval stage, exposure time, assay protocol, mortality end point, and concentration units. Accordingly, the reported values are not directly comparable and should not be used to establish a quantitative ranking of larvicidal potency. NR = not reported.

## 4 Conclusion

In summary, a rutin–Zn material was prepared using a nominal 1:1 Zn:rutin precursor molar ratio and characterized by complementary spectroscopic, diffraction, and DLS techniques. UV–Vis, FTIR, and NMR results support an interaction between Zn^2+^ and oxygenated regions of rutin, with changes in its electronic and local chemical environments, although no unique coordination site, molecular geometry, or ligand-to-metal stoichiometry could be established. Powder XRD indicated a heterogeneous product containing residual crystalline precursors and an X-ray-amorphous fraction, without evidence of a new crystalline rutin–Zn phase. DLS showed that reaction with Zn^2+^ altered the aggregation behavior of rutin, producing larger solvated aggregates; therefore, the reported hydrodynamic diameters should not be interpreted as primary particle sizes or solid-state structural dimensions. The rutin–Zn material exhibited a stronger concentration–mortality response than free rutin against *Aedes aegypti* larvae, although its absolute activity remained moderate. Because zinc acetate and physical-mixture controls were not included, the respective contributions of Zn^2+^–rutin interaction, residual zinc species, and combined exposure could not be distinguished. Overall, the results provide preliminary physicochemical and biological evidence that reaction with Zn^2+^ modifies rutin and is associated with improved larvicidal performance under the tested laboratory conditions.
